# High vs. low radiotherapy dose in locally advanced esophageal squamous cell carcinoma patients treated with neoadjuvant concurrent chemoradiotherapy: an endemic area population-based study

**DOI:** 10.1007/s12672-022-00594-y

**Published:** 2022-11-24

**Authors:** Chia-Chin Li, Chih-Yi Chen, Ying-Hsiang Chou, Chih-Jen Huang, Hsiu-Ying Ku, Ying-Chun Lin, Chun-Ru Chien

**Affiliations:** 1 Department of Radiation Oncology, China Medical University Hospital, Taichung, Taiwan; 2 Division of Thoracic Surgery, Department of Surgery, Chung Shan Medical University Hospital, Taichung, Taiwan; 3 Institute of Medicine, Chung Shan Medical University, Taichung, Taiwan; 4 Department of Medical Imaging and Radiological Sciences, Chung Shan Medical University, Taichung, Taiwan; 5 Department of Radiation Oncology, Chung Shan Medical University Hospital, Taichung, Taiwan; 6 Department of Radiation Oncology, Kaohsiung Medical University Hospital, Kaohsiung, Taiwan; 7 National Institute of Cancer Research, National Health Research Institutes Miaoli, Miaoli County, Taiwan; 8 Department of Healthcare Administration, Asia University, Taichung, Taiwan; 9 School of Medicine, College of Medicine, China Medical University, No. 91 Hsueh-Shih Road, North District, Taichung, 40402 Taiwan

**Keywords:** Esophageal squamous cell carcinoma, Neoadjuvant concurrent chemoradiotherapy, Radiotherapy dose

## Abstract

**Background:**

The optimal radiotherapy dose for locally advanced esophageal squamous cell carcinoma in endemic areas treated with neoadjuvant concurrent chemoradiotherapy is unclear.

**Methods:**

Eligible patients diagnosed between 2010 and 2019 were identified via the Taiwan Cancer Registry. We used propensity score (PS) weighting to balance observable potential confounders. The hazard ratio (HR) of death was compared between high dose (50–50.4 Gy) and low dose (40–41.4 Gy) radiotherapy. We also evaluated other outcomes and performed supplementary analyses via an alternative approach.

**Results:**

Our study population consisted of 644 patients. The PS weight-adjusted HR of death was 0.92 (95% confidence interval: 0.7–1.19, p = 0.51). There were no statistically significant differences for other outcomes or supplementary analyses.

**Conclusions:**

In this population-based study from an endemic area, we found no significant difference in overall survival between high vs. low radiotherapy doses.

**Supplementary Information:**

The online version contains supplementary material available at 10.1007/s12672-022-00594-y.

## Background

Esophageal cancer is a common cause of cancer mortality around the world [1]. The common histological types are squamous cell carcinoma in the East and adenocarcinoma in the West [1, 2]. For locally advanced esophageal squamous cell carcinoma (LA-ESCC) patients, neoadjuvant concurrent chemoradiotherapy (nCCRT) is one of the most commonly recommended approaches in the literature [1, 3–5].

However, the optimal radiotherapy (RT) dose of nCCRT for ESCC is unclear. In the current national comprehensive cancer network (NCCN) guidelines, a wide range of doses (41.4–50.4 Gy) are recommended [3]. Relatively low doses (40–41.4 Gy) were used in two landmark randomized controlled trials (RCTs) [6–8]. One may speculate whether a higher dose may lead to better outcomes due to the commonly believed radiotherapy dose response [9]. However, a systematic review (SR) published in 2021 reported that a low radiotherapy dose using modern techniques might provide the optimal therapeutic ratio for esophageal cancer patients treated with nCCRT [10]. Similar results were obtained in ESCC subgroup analyses [10]. However, only three non-RCTs included in this SR [10] directly compared high vs. low doses [11–13]. This systematic review [10] (including studies not limited to RCTs) also commented on results from three population-based studies [14–16] and stated that a dose of 40.0–41.4 Gy did not negatively impact overall survival (OS) compared to a higher dose. Another systematic review (including only RCTs comparing nCCRT vs. surgery) published in 2019 reported no difference in OS between low-dose and high-dose radiotherapy [17].

All of the abovementioned studies [11–16] that directly compared high vs. low radiotherapy doses were non-RCTs conducted in North America or Europe rather than an endemic area of ESCC [1, 2]. Due to an insufficiency of RT dose-relevant studies, as mentioned above, and since the benefit of nCCRT is possibly larger for ESCC than for adenocarcinoma, as reported in the long-term results of the CROSS study [18], we aimed to directly compare high vs. low RT doses in nCCRT for LA-ESCC patients from Taiwan, an endemic area of ESCC [2].

## Materials and methods

### Data source

In this retrospective cohort study, we used the Taiwan Cancer Registry (TCR) with personal identifiers removed for our data analysis. The TCR database provides comprehensive information (such as patient, disease, and treatment characteristics) and has been reported to be one of the highest quality cancer registries in the world [19, 20].

### Study design, study population, and intervention

**Fig. 1:**
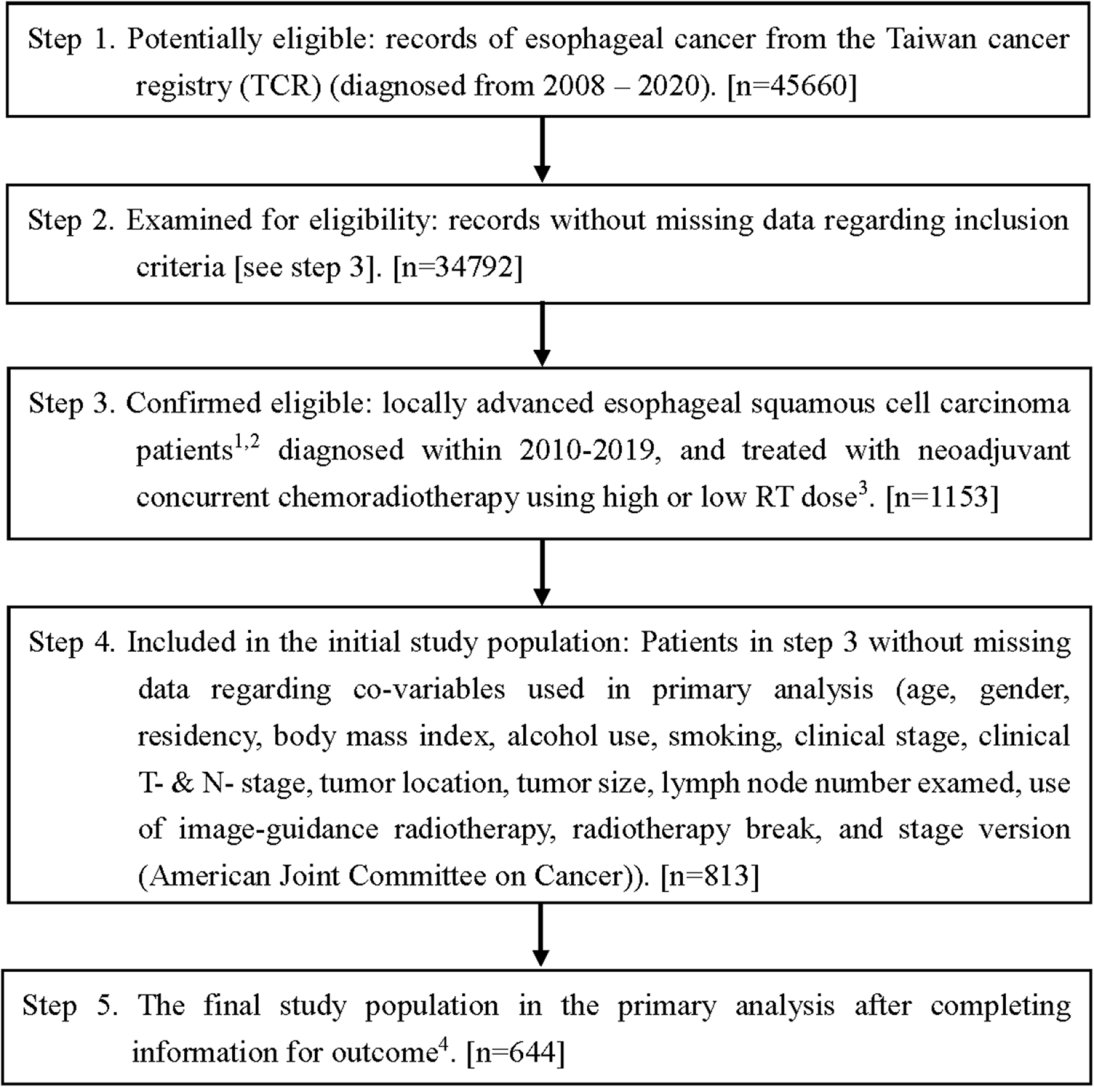
STROBE study flowchart and the number of individuals at each stage of the study. ^1^: We only included those treated (class 1–2) to ensure data consistency. ^2^: Clinical stage cT2-4aN0M0 or cT1-4aN1-3M0 from the 7th or 8th American Joint Committee on Cancer staging manual. ^3^: High RT dose (50 Gy/25 fractions or 50.4 Gy/28 fractions), low RT dose (40 Gy/20 fractions or 41.4 Gy/23 fractions). ^4^: Without missing information in the TCR and death registry regarding survival status and cause of death

The study flowchart as suggested in the STROBE statement [21] is depicted in Fig. 1. We identified LA-ESCC adult patients diagnosed from 2010–2019 and treated with nCCRT followed by esophagectomy within 4–12 weeks after RT. We did not include patients diagnosed before 2010 because the T/N/M staging in the American Joint Committee on Cancer (AJCC) cancer staging manual 7th edition (used since 2010) was different from the AJCC 6th edition but the same as the AJCC 8th edition [22, 23]. Locally advanced stage was defined as clinical stage cT2-4aN0M0 or cT1-4aN1-3M0 by the AJCC 7th or 8th edition [22, 23]. We included adult patients aged 18–75 years who were treated with modern (3D conformal or intensity modulated) radiotherapy techniques according to the literature [10] and excluded those with multiple treatment records or prior cancer(s) to ensure data quality. We further selected patients treated with nCCRT using a high external beam radiotherapy dose (50 Gy/25 fractions or 50.4 Gy/28 fractions) or a low RT dose (40 Gy/20 fractions or 41.4 Gy/23 fractions) according to the literature [3, 6, 10, 18].

Our explanatory variable of interest (high vs. low RT dose), primary outcome of interest (OS) and other supplementary outcomes (incidence of esophageal cancer mortality (IECM), pathological complete response (pCR) and R0 resection (no invasive cancer at surgical margin)) were determined via recordings in the TCR or death registry. We also defined the date of diagnosis as the index date and calculated the OS or IECM from the index date to the date of death or to Dec 31, 2020 (the censoring date of the death registry).

### Covariates

We collected covariates to adjust for potential nonrandomized treatment selection. These covariates were modified from recent relevant studies and our clinical and research experience [6, 7, 24, 25]. Patient demographics (age, gender, residency), patient characteristics (body mass index (BMI), alcohol use, smoking), disease characteristics (clinical stage and version, clinical T- and N- stage, tumor location, tumor size, number of lymph nodes (LNs) examined), and treatment characteristics (use of image-guided radiotherapy (IGRT), radiotherapy break) were defined as follows. The patient residency region was classified as northern Taiwan or nonnorth. Smoking, alcohol use and the use of IGRT were classified as yes or no. The clinical stage was classified as 1–2 or 3–4A. The clinical T-stage was classified as 1–2 or 3–4. The clinical N-stage was classified as 0 or 1–3. Tumor location was classified as cervical vs. noncervical. Tumor size was classified by a diameter <  = 5 cm or > 5 cm. Those with radiotherapy prolongation of more than one week were classified as yes for a radiotherapy break, whereas those without were classified as no. Cancer staging manual version was classified according to the AJCC 7th or 8th edition. The other covariates (age, BMI, number of LNs examined) were defined as continuous variables.

### Statistical analysis

As advocated in the literature [26–29], we adopted the propensity score (PS) approach to balance the measured potential confounders and used PS weighting (PSW) as the primary framework for analysis [30].

In the primary analysis (PA), we evaluated the probability of receiving a high RT dose (vs. a low RT dose) as the PS via a logistic regression model based on the above covariates and then assessed the balance in covariates between groups after PSW using the overlap weight [30] via the standardized difference (SDif). During the entire follow-up period, we compared the hazard ratio (HR) of death between groups via the Cox proportional hazards model in the weighted sample for point estimation and used the bootstrap method to estimate the 95% confidence interval (95% CI) [31–33]. We used the E-value to assess the robustness of our findings regarding potential unmeasured confounder(s), as suggested in the literature [34]. We also evaluated the IECM between groups using the competing risk approach [35] and compared pCR and R0 resection between groups in the weighted sample [36].

In the supplementary analysis (SA), we performed seven analyses during revision to clarify the robustness of our findings. In SA-1, we used an alternative analytic approach (PS matching, PSM) for the primary study population by constructing a subgroup (1:1 PS matched cohort without replacement) and then compared the HR of death (via a robust variance estimator [31]) and other outcomes [37, 38] between groups. In SA-2, we explored the impact of the radiotherapy dose on additional outcomes (disease-free survival (DFS), local regional recurrence free survival (LRRFS), distant metastatic free survival (DMFS), and peri-operative mortality (POM)) among patients without missing information in the TCR. We used postoperative 30-day mortality as POM per the literature [39]. In SA-3 ~ SA-6, we performed subgroup analyses for patients with cT1-2, cT3-4, cN0, and cN1-3 disease. In SA-7, we performed the analyses among patients with additional information regarding surgical method (with or without minimally invasive esophagectomy, MIE) available in the TCR.

The statistical analyses were performed using SAS 9.4 software (SAS Institute, Cary, NC) and R version 4.2.0 (R Development Core Team, R Foundation for Statistical Computing, Vienna, Austria).

## Results

### Study population

As shown in Fig. 1, the study population consisted of 644 eligible LA-ESCC adult patients who received a high RT dose (430 patients) or a low RT dose (214 patients) from 2010 to 2019. The patient characteristics are described in Table 1. Some covariates (age, residency, number of LNs examined, clinical stage, clinical T stage, RT break, and staging manual version) were imbalanced before PS weighting [40], but all covariates achieved balance (standardized differences ≈ 0) after PS weighting via the overlap weights.

**Table 1:** Patient characteristics of the study population in the primary analysis

	High RT dose (n = 430)		Low RT dose (n = 214)		Standardized difference^a^	
	Number or mean (SD)^a^	(%)^a^	Number or mean (SD)^a^	(%)^a^	Before PSW	After PSW
Age	54.98 (7.98)		57.25 (8.45)		0.276	≈ 0
Gender						
Female	26	(6)	13	(6)	0.001	≈ 0
Male	404	(94)	201	(94)		
Residency						
Nonnorth	303	(70)	58	(27)	0.963	≈ 0
North	127	(30)	156	(73)		
BMI	22.30 (3.61)		22.43 (3.38)		0.037	≈ 0
Alcohol use						
No	60	(14)	39	(18)	0.116	≈ 0
Yes	370	(86)	175	(82)		
Smoking						
No	55	(13)	29	(14)	0.022	≈ 0
Yes	375	(87)	185	(86)		
Clinical stage						
1–2	84	(20)	19	(9)	0.309	≈ 0
3–4A	346	(80)	195	(91)		
Clinical T-stage						
T1–T2	64	(15)	10	(5)	0.349	≈ 0
T3–T4	366	(85)	204	(95)		
Clinical N-stage						
N0	53	(12)	14	(7)	0.199	≈ 0
N1–N3	377	(88)	200	(93)		
Tumor location						
Noncervical	428	(99)	211	(99)	0.098	≈ 0
Cervical	2	(1)	3	(1)		
Tumor size						
≤ 5 cm	202	(47)	101	(47)	0.004	≈ 0
> 5 cm	228	(53)	113	(53)		
Number of LNs examined	22.44 (12.65)		35.67 (17.61)		0.863	≈ 0
Use of IGRT						
No	338	(79)	155	(72)	0.144	≈ 0
Yes	92	(21)	59	(28)		
RT break						
≤ 1 week	401	(93)	211	(99)	0.273	≈ 0
> 1 week	29	(7)	3	(1)		
AJCC staging manual version						
7th edition	301	(70)	181	(85)	0.353	≈ 0
8th edition	129	(30)	33	(15)		

*AJCC* American Joint Committee on Cancer, *BMI* body mass index, *IGRT* image-guided radiotherapy, *LN* lymph node, *PSW* propensity score weighting, *RT* radiotherapy, *SD* standard deviation

^a^Rounded

### Primary analysis

**Fig. 2:**
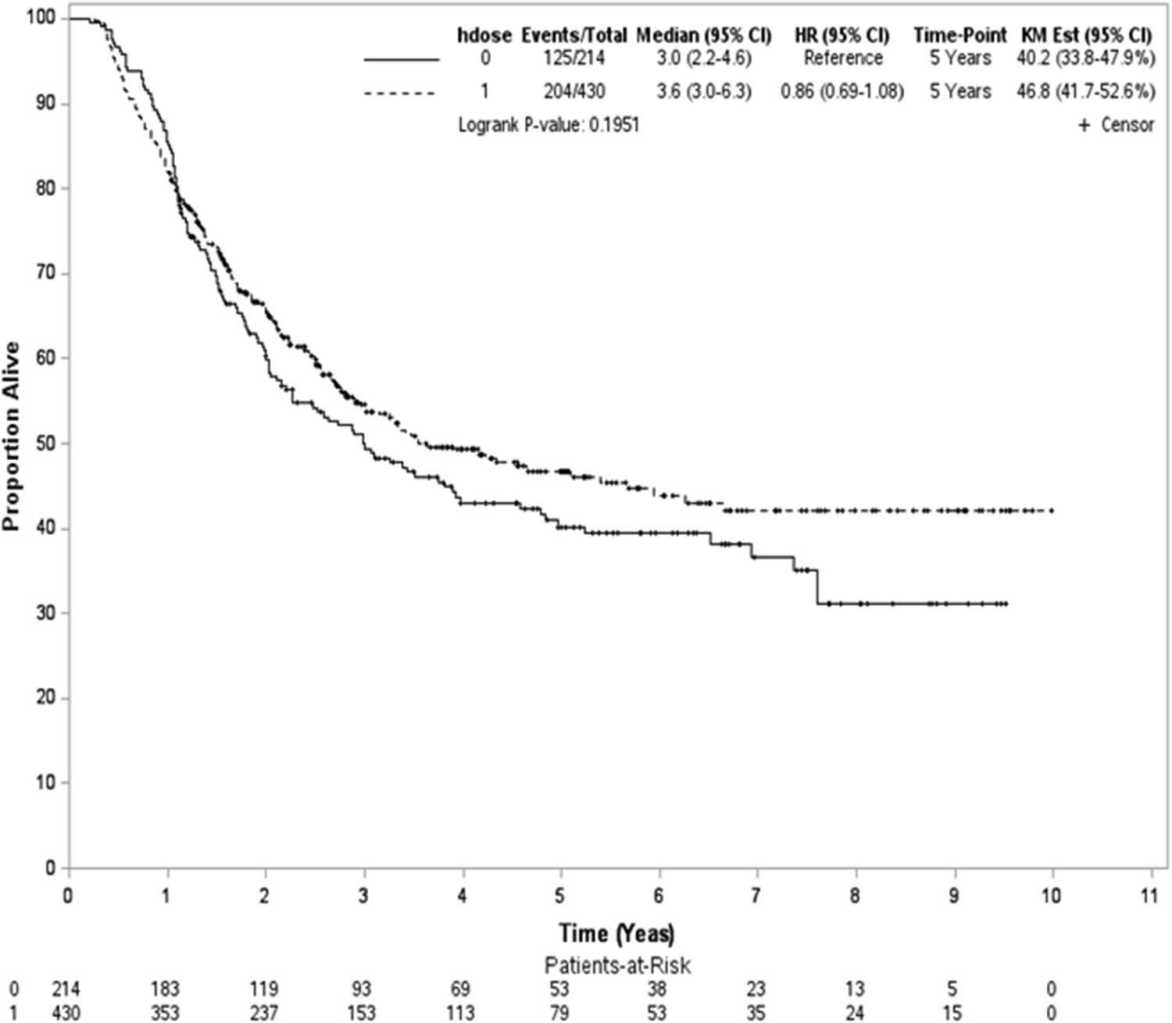
Kaplan–Meier unadjusted overall survival curve (in years) in the primary analysis. *hdose* high dose

**Fig. 3:**
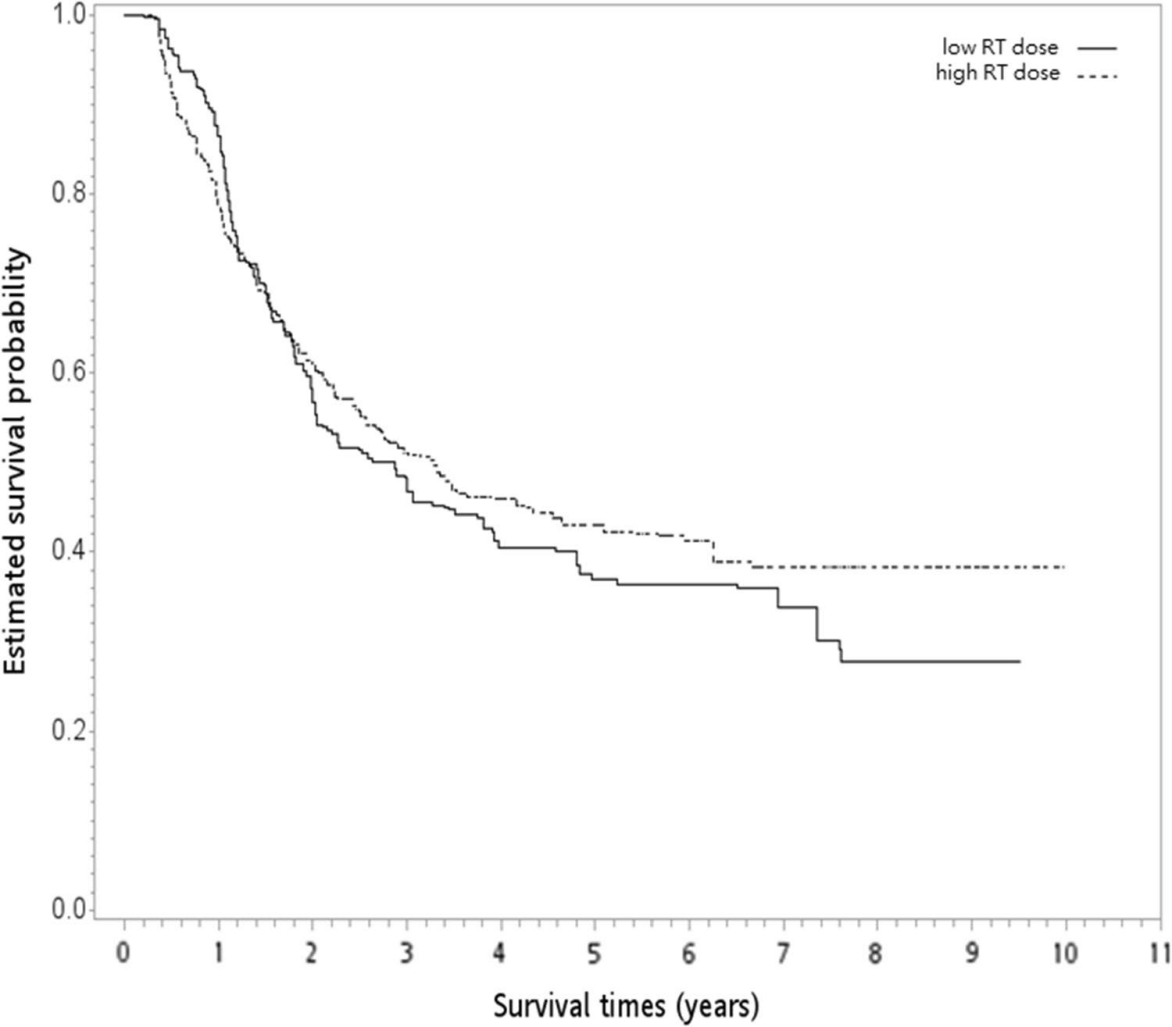
The overlap weight-adjusted overall survival curve (in years) in the primary analysis. *RT* radiotherapy

After a median follow-up of 27 months (range 3–119 months), 329 deaths were observed (204 and 125 patients for the high and low RT dose groups, respectively). The median follow-up was 49 months (range 12–119 months) for survivors. In the unadjusted analysis, the 5-year OS rates were 47% and 40% for the high and low RT dose groups, respectively (log-rank test, P = 0.2; Fig. 2). The overlap weight-adjusted OS curve is shown in Fig. 3. The 5-year PSW-adjusted OS rates for the two groups were 43% (high RT dose) and 37% (low RT dose). The PSW-adjusted HR of death was 0.92 (95% CI 0.70–1.19, P = 0.51) when a high RT dose was compared to a standard RT dose. The observed HR of 0.92 for OS could be explained by an unmeasured confounder associated with the selection of treatment (high or low RT dose) and survival by a risk ratio of 1.31 (E-value)-fold each, but weaker confounding factors could not. The result was not significantly different for the IECM (HR = 0.98, P = 0.93). We also found that the rates of pCR and R0 resection were not significantly different between the groups. The PSW-adjusted relative risks were 1.11 (95% CI 0.90–1.33) for pCR and 1 (95% CI 0.96–1.04) for R0 resection.

### Supplementary analysis

**Fig. 4:**
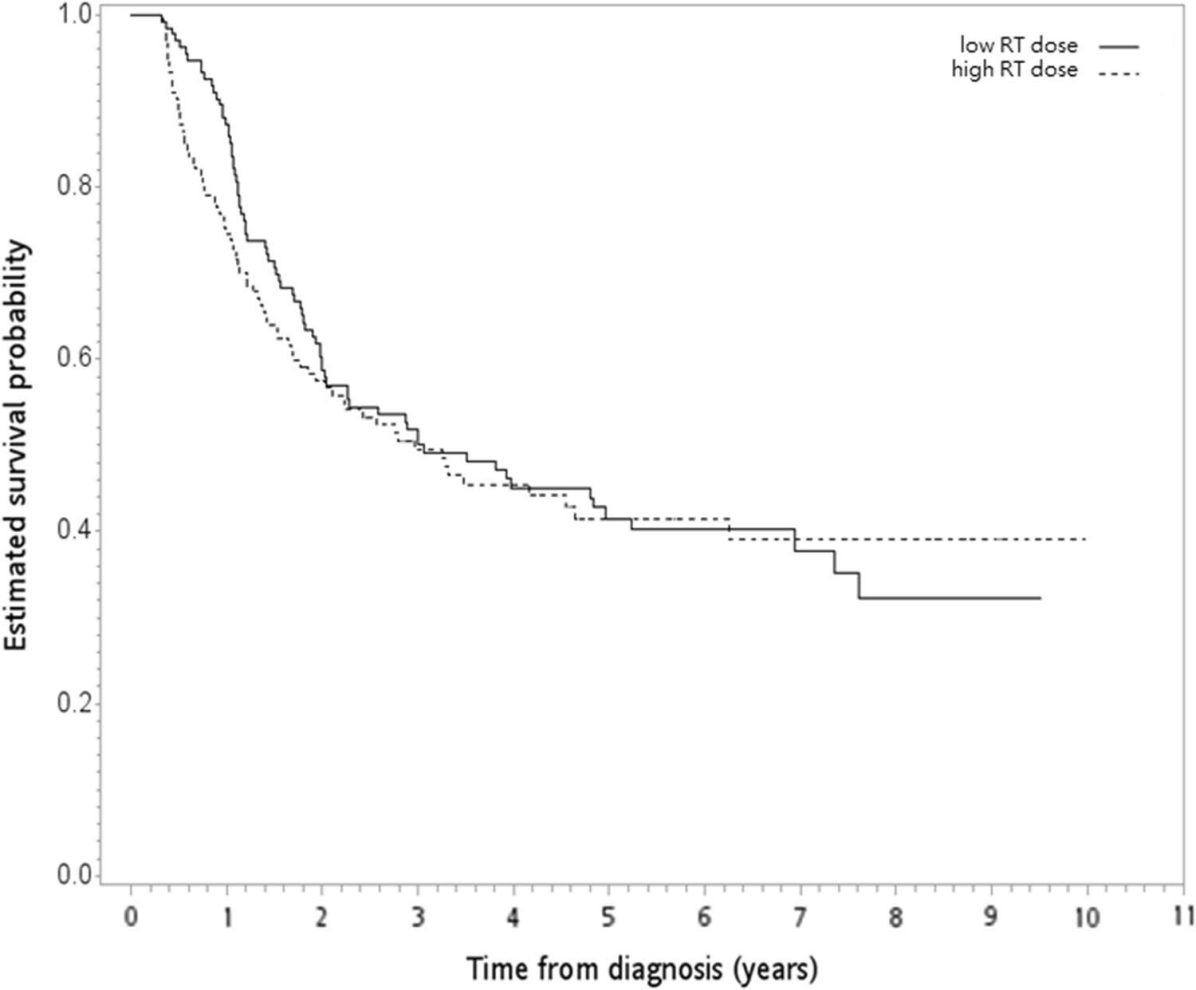
Kaplan‒Meier overall survival curve (in years) in supplementary analysis 1. *RT* radiotherapy

The constructed PS-matched subgroup (n = 268) for SA-1 is shown in Table 2, and all covariates were balanced after PSM [40]. The Kaplan–Meier OS curve is shown in Fig. 4. The 5-year OS rates were 42% (high RT dose) and 42% (low RT dose), and there was no statistically significant difference between the groups (HR = 1.07, 95% CI 0.77–1.49, P = 0.68). The results were not significant for the IECM (HR = 1.05; 95% CI = 0.73–1.52, p = 0.79). We also found that the rates of pCR and R0 resection were not significantly different between the groups (P = 1 for both pCR and R0). In SA-2, there were 600 patients without missing information in the TCR regarding recurrence with a good balance of covariates after PSW (Table S1). We found no significant differences in DFS (HR = 0.96, P = 0.77), LRRFS (HR = 0.77, P = 0.14), or DMFS (HR = 1.08, P = 0.33). The rate of POM was also similar between the two groups, with a PSW-adjusted relative risk of 0.56 (P = 0.21). In SA-3, among patients with cT1-2 disease, we found that the distribution of covariates between the groups was balanced after PSW (Table S2), and the PSW-adjusted HR of death was 1.64 (P = 0.47) when a high RT dose was compared to a standard RT dose. In SA-4, SA-5 and SA-6, the distribution of covariates between the groups was also balanced after PSW (Tables S3, S4 and S5), and the corresponding results were 0.89 (P = 0.42), 0.44 (P = 0.64), and 0.99 (P = 0.94) for patients with cT3-4, cN0, and cN1-3 disease, respectively. In SA-7, among patients with additional information regarding the surgical method, the distribution of covariates (including the surgical method) between the groups was also balanced after PSW (see Table S6), and the PSW-adjusted HR of death was 1.10 (P = 0.28) when a high RT dose was compared to a standard RT dose.

**Table 2:** Patient characteristics of the PS-matched subgroup

	High RT dose (n = 134)		Low RT dose (n = 134)		
	Number or mean (SD)^a^	(%)^a^	Number or mean (SD)^a^	(%)^a^	Standardized difference^a^
Age	56.54 (8.55)		56.30 (8.36)		0.028
Gender					
Female	9	(7)	6	(4)	0.098
Male	125	(93)	128	(96)	
Residency					
Nonnorth	63	(47)	55	(41)	0.120
North	71	(53)	79	(59)	
BMI	21.89 (3.74)		22.49 (3.41)		0.169
Alcohol use					
No	24	(18)	24	(18)	0
Yes	110	(82)	110	(82)	
Smoking					
No	20	(15)	19	(14)	0.021
Yes	114	(85)	115	(86)	
Clinical stage					
1–2	20	(15)	15	(11)	0.111
3–4A	114	(85)	119	(89)	
Clinical T-stage					
T1–T2	10	(7)	9	(7)	0.029
T3–T4	124	(93)	125	(93)	
Clinical N-stage					
N0	14	(10)	11	(8)	0.077
N1–N3	120	(90)	123	(92)	
Tumor location					
Noncervical	134	(100)	134	(100)	
Cervical	0	(0)	0	(0)	
Tumor size					
≤ 5 cm	62	(46)	60	(45)	0.030
> 5 cm	72	(54)	74	(55)	
Number of LNs examined	28.13 (14.92)		27.53 (13.80)		0.042
Use of IGRT					
No	100	(75)	101	(75)	0.017
Yes	34	(25)	33	(25)	
RT break					
≤ 1 week	129	(96)	131	(98)	0.088
> 1 week	5	(4)	3	(2)	
AJCC staging manual version					
7th edition	109	(81)	107	(80)	0.038
8th edition	25	(19)	27	(20)	

*AJCC* American Joint Committee on Cancer, *BMI* body mass index, *IGRT* image-guided radiotherapy, *LN* lymph node, *PSW* propensity score weighting, *RT* radiotherapy, *SD* standard deviation

^a^Rounded

## Discussion

In this population-based study, we found no significant difference in OS between high vs. low RT doses, and there were no significant differences between other endpoints. To our knowledge, this was the first population-based study from an endemic area of ESCC.

The results of our study were in line with the results from a previous systematic review published in 2019 [17]. The lack of an obvious radiotherapy dose response in this dose range may be in line with the lack of a radiotherapy dose response within 50–60 Gy in definitive concurrent chemoradiotherapy for esophageal cancer [41–43]. Another possibility was that although there was a radiotherapy dose response, the clinical benefit was diminished after subsequent esophagectomy. A similar possibility has been reported in rectal cancer patients treated with neoadjuvant therapies [44]. Therefore, the use of a low dose in current clinical practice seems reasonable for patients deemed to receive esophagectomy. However, the optimal dose may be unclear in the era of adjuvant immunotherapy [45, 46].

There were several limitations of the current study. First, potential unobservable covariates are always possible in nonrandomized studies, although we used the propensity score method to adjust for observable covariates. Therefore, some factors, such as performance status, chemotherapy regimen and definition of radiotherapy volume, which are not the same for every patient, were not included in our analyses due to data limitations. However, we reported the E-value to evaluate the robustness of our findings to potential unobservable covariates, as suggested in the literature [34]. Second, the use of postrecurrence therapy (such as immunotherapy [47]) may have impacted our primary endpoint (OS) but could not be evaluated due to data limitations in the TCR. Finally, other endpoints, such as quality of life and peri-operative or late complications (all were likely to be exacerbated by high-dose radiotherapy), in addition to our primary endpoint (OS), might also be relevant, but these were not investigated due to concerns regarding data availability.

## Conclusions

In this population-based study from an endemic area, we found no significant difference in OS between high vs. low radiotherapy doses for locally advanced esophageal squamous cell carcinoma patients treated with neoadjuvant concurrent chemoradiotherapy followed by esophagectomy. Further studies are needed to clarify our findings.

## Supplementary Information

Additional file1 (DOCX 81 KB)

## Data Availability

The data that support the findings of this study are available from the Taiwan Cancer Registry Center, but restrictions apply to the availability of these data, which were used under license for the current study and are not publicly available. Data are, however, available from the authors upon reasonable request and with permission of the Taiwan Cancer Registry Center.

## Abbreviations

95% CI: 95% Confidence interval
AJCC: American Joint Committee on Cancer
BMI: Body mass index
HR: Hazard ratio
IECM: Incidence of esophageal cancer mortality
IGRT: Image-guided radiotherapy
LA-ESCC: Locally advanced esophageal squamous cell carcinoma
LN: Lymph node
nCCRT: Neoadjuvant concurrent chemoradiotherapy
OS: Overall survival
pCR: Pathological complete response
PA: Primary analysis
PS: Propensity score
PSM: PS matching
PSW: PS weighting
RCT: Randomized controlled trial
RT: Radiotherapy
SA: Supplementary analysis
SDif: Standardized difference
SR: Systematic review
TCR: Taiwan Cancer Registry
