# Supplementary material for: High vs. low radiotherapy dose in locally advanced esophageal squamous cell carcinoma patients treated with neoadjuvant concurrent chemoradiotherapy: an endemic area population-based study
Source: Discov Oncol. 2022 Nov 24;13:130. doi: 10.1007/s12672-022-00594-y (PMC9700542; doi:10.1007/s12672-022-00594-y)
Supplement: Supplementary file 1 — Additional file1 (DOCX 81 KB) [file 12672_2022_594_MOESM1_ESM.docx]

Table S1. Patient characteristics of the study population in SA-2

|  |  | High RT dose  (n=395) | | Low RT dose  (n=205) | | Standardized difference^†^ | |
| --- | --- | --- | --- | --- | --- | --- | --- |
|  |  | Number or mean (SD)^†^ | (%)^†^ | Number or mean (SD)^†^ | (%)^†^ | Before  PSW | After  PSW |
| Age |  | 54.95 (8.07) |  | 57.40 (8.54) |  | 0.295 | ≈ 0 |
| Gender | Female | 25 | (6) | 13 | (6) | 0.001 | ≈ 0 |
|  | Male | 370 | (94) | 192 | (94) |  |  |
| Residency | Nonnorth | 282 | (71) | 57 | (28) | 0.969 | ≈ 0 |
|  | North | 113 | (29) | 148 | (72) |  |  |
| BMI |  | 22.34 (3.63) |  | 22.38 (3.38) |  | 0.012 | ≈ 0 |
| Alcohol use | No | 54 | (14) | 38 | (19) | 0.133 | ≈ 0 |
|  | Yes | 341 | (86) | 167 | (81) |  |  |
| Smoking | No | 48 | (12) | 29 | (14) | 0.059 | ≈ 0 |
|  | Yes | 347 | (88) | 176 | (86) |  |  |
| Clinical stage | 1–2 | 80 | (20) | 19 | (9) | 0.313 | ≈ 0 |
|  | 3–4A | 315 | (80) | 186 | (91) |  |  |
| Clinical T-stage | T1–T2 | 60 | (15) | 10 | (5) | 0.348 | ≈ 0 |
|  | T3–T4 | 335 | (85) | 195 | (95) |  |  |
| Clinical N-stage | N0 | 49 | (12) | 14 | (7) | 0.190 | ≈ 0 |
|  | N1-N3 | 346 | (88) | 191 | (93) |  |  |
| Tumor location | Noncervical | 393 | (99) | 202 | (99) | 0.097 | ≈ 0 |
|  | Cervical | 2 | (1) | 3 | (1) |  |  |
| Tumor size | ≤5 cm | 188 | (48) | 98 | (48) | 0.004 | ≈ 0 |
|  | > 5 cm | 207 | (52) | 107 | (52) |  |  |
| Number of LNs examined |  | 22.47 (12.76) |  | 35.41 (17.52) |  | 0.844 | ≈ 0 |
| Use of IGRT | No | 314 | (79) | 149 | (73) | 0.160 | ≈ 0 |
|  | Yes | 81 | (21) | 56 | (27) |  |  |
| RT break | ≤1 week | 367 | (93) | 202 | (99) | 0.281 | ≈ 0 |
|  | >1 week | 28 | (7) | 3 | (1) |  |  |
| AJCC staging manual version | 7^th^ edition | 274 | (69) | 174 | (85) | 0.376 | ≈ 0 |
|  | 8^th^ edition | 121 | (31) | 31 | (15) |  |  |

*AJCC* American Joint Committee on Cancer, *BMI* body mass index, *IGRT* image-guided radiotherapy, *LN* lymph node, *PSW* propensity score weighting, *RT* radiotherapy, *SD* standard deviation.

^†^Rounded.

Table S2. Patient characteristics of the cT1-2 subgroup in SA-3

|  |  | High RT dose  (n=64) | | Low RT dose  (n=10) | | Standardized difference^†^ | |
| --- | --- | --- | --- | --- | --- | --- | --- |
|  |  | Number or mean (SD)^†^ | (%)^†^ | Number or mean (SD)^†^ | (%)^†^ | Before  PSW | After  PSW |
| Age |  | 54.92 (7.77) |  | 62.30 (8.35) |  | 0.915 | ≈ 0 |
| Gender | Female | 6 | (9) | 0 | (0) | 0.455 | ≈ 0 |
|  | Male | 58 | (91) | 10 | (100) |  |  |
| Residency | Nonnorth | 40 | (62) | 4 | (40) | 0.462 | ≈ 0 |
|  | North | 24 | (38) | 6 | (60) |  |  |
| BMI |  | 23.22 (3.29) |  | 24.09 (3.35) |  | 0.263 | ≈ 0 |
| Alcohol use | No | 8 | (13) | 4 | (40) | 0.658 | ≈ 0 |
|  | Yes | 56 | (87) | 6 | (60) |  |  |
| Smoking | No | 12 | (19) | 1 | (10) | 0.251 | ≈ 0 |
|  | Yes | 52 | (81) | 9 | (90) |  |  |
| Clinical stage | 1–2 | 42 | (66) | 6 | (60) | 0.117 | ≈ 0 |
|  | 3–4A | 22 | (34) | 4 | (40) |  |  |
| Clinical N-stage | N0 | 10 | (16) | 1 | (10) | 0.169 | ≈ 0 |
|  | N1-N3 | 54 | (84) | 9 | (90) |  |  |
| Tumor location | Noncervical | 10 | (100) | 64 | (100) | . | . |
|  | Cervical | 0 | (0) | 0 | (0) |  |  |
| Tumor size | ≤5 cm | 43 | (67) | 8 | (80) | 0.294 | ≈ 0 |
|  | > 5 cm | 21 | (33) | 2 | (20) |  |  |
| Number of LNs examined |  | 25.98 (12.14) |  | 22.80 (12.66) |  | 0.257 | ≈ 0 |
| Use of IGRT | No | 46 | (72) | 6 | (60) | 0.253 | ≈ 0 |
|  | Yes | 18 | (28) | 4 | (40) |  |  |
| RT break | ≤1 week | 58 | (91) | 10 | (100) | 0.455 | ≈ 0 |
|  | >1 week | 6 | (9) | 0 | (0) |  |  |
| AJCC staging manual version | 7^th^ edition | 53 | (83) | 6 | (60) | 0.522 | ≈ 0 |
|  | 8^th^ edition | 11 | (17) | 4 | (40) |  |  |

*AJCC* American Joint Committee on Cancer, *BMI* body mass index, *IGRT* image-guided radiotherapy, *LN* lymph node, *PSW* propensity score weighting, *RT* radiotherapy, *SD* standard deviation.

^†^Rounded.

Table S3. Patient characteristics of the cT3-4 subgroup in SA-4

|  |  | High RT dose  (n=366) | | Low RT dose  (n=204) | | Standardized difference^†^ | |
| --- | --- | --- | --- | --- | --- | --- | --- |
|  |  | Number or mean (SD)^†^ | (%)^†^ | Number or mean (SD)^†^ | (%)^†^ | Before  PSW | After  PSW |
| Age |  | 54.99 (8.03) |  | 57.00 (8.40) |  | 0.245 | ≈ 0 |
| Gender | Female | 20 | (5) | 13 | (6) | 0.038 | ≈ 0 |
|  | Male | 346 | (95) | 191 | (94) |  |  |
| Residency | Nonnorth | 263 | (72) | 54 | (26) | 1.019 | ≈ 0 |
|  | North | 103 | (28) | 150 | (74) |  |  |
| BMI |  | 22.14 (3.64) |  | 22.35 (3.36) |  | 0.059 | ≈ 0 |
| Alcohol use | No | 52 | (14) | 35 | (17) | 0.081 | ≈ 0 |
|  | Yes | 314 | (86) | 169 | (83) |  |  |
| Smoking | No | 43 | (12) | 28 | (14) | 0.059 | ≈ 0 |
|  | Yes | 323 | (88) | 176 | (86) |  |  |
| Clinical stage | 1–2 | 42 | (11) | 13 | (6) | 0.180 | ≈ 0 |
|  | 3–4A | 324 | (89) | 191 | (94) |  |  |
| Clinical N-stage | N0 | 43 | (12) | 13 | (6) | 0.188 | ≈ 0 |
|  | N1-N3 | 323 | (88) | 191 | (94) |  |  |
| Tumor location | Noncervical | 364 | (99) | 201 | (99) | 0.093 | ≈ 0 |
|  | Cervical | 2 | (1) | 3 | (1) |  |  |
| Tumor size | ≤5 cm | 159 | (43) | 93 | (46) | 0.043 | ≈ 0 |
|  | > 5 cm | 207 | (57) | 111 | (54) |  |  |
| Number of LNs examined |  | 21.83 (12.65) |  | 36.30 (17.60) |  | 0.944 | ≈ 0 |
| Use of IGRT | No | 292 | (80) | 149 | (73) | 0.159 | ≈ 0 |
|  | Yes | 74 | (20) | 55 | (27) |  |  |
| RT break | ≤1 week | 343 | (94) | 201 | (99) | 0.251 | ≈ 0 |
|  | >1 week | 23 | (6) | 3 | (1) |  |  |
| AJCC staging manual version | 7^th^ edition | 248 | (68) | 175 | (86) | 0.437 | ≈ 0 |
|  | 8^th^ edition | 118 | (32) | 29 | (14) |  |  |

*AJCC* American Joint Committee on Cancer, *BMI* body mass index, *IGRT* image-guided radiotherapy, *LN* lymph node, *PSW* propensity score weighting, *RT* radiotherapy, *SD* standard deviation.

^†^Rounded.

Table S4. Patient characteristics of the cN0 subgroup in SA-5

|  |  | High RT dose  (n=53) | | Low RT dose  (n=14) | | Standardized difference^†^ | |
| --- | --- | --- | --- | --- | --- | --- | --- |
|  |  | Number or mean (SD)^†^ | (%)^†^ | Number or mean (SD)^†^ | (%)^†^ | Before  PSW | After  PSW |
| Age |  | 54.13 (8.22) |  | 59.36 (9.36) |  | 0.593 | ≈ 0 |
| Gender | Female | 3 | (6) | 1 | (7) | 0.061 | ≈ 0 |
|  | Male | 50 | (94) | 13 | (93) |  |  |
| Residency | Nonnorth | 38 | (72) | 4 | (29) | 0.956 | ≈ 0 |
|  | North | 15 | (28) | 10 | (71) |  |  |
| BMI |  | 22.35 (3.81) |  | 20.27 (3.26) |  | 0.587 | ≈ 0 |
| Alcohol use | No | 5 | (9) | 4 | (29) | 0.503 | ≈ 0 |
|  | Yes | 48 | (91) | 10 | (71) |  |  |
| Smoking | No | 8 | (15) | 3 | (21) | 0.165 | ≈ 0 |
|  | Yes | 45 | (85) | 11 | (79) |  |  |
| Clinical stage | 1–2 | 52 | (98) | 14 | (100) | 0.196 | ≈ 0 |
|  | 3–4A | 1 | (2) | 0 | (0) |  |  |
| Clinical T-stage | T1–T2 | 10 | (19) | 1 | (7) | 0.354 | ≈ 0 |
|  | T3–T4 | 43 | (81) | 13 | (93) |  |  |
| Tumor location | Noncervical | 53 | (100) | 14 | (100) | . | . |
|  | Cervical | 0 | (0) | 0 | (0) |  |  |
| Tumor size | ≤5 cm | 27 | (51) | 8 | (57) | 0.125 | ≈ 0 |
|  | > 5 cm | 26 | (49) | 6 | (43) |  |  |
| Number of LNs examined |  | 18.75 (12.83) |  | 27.79 (17.08) |  | 0.598 | ≈ 0 |
| Use of IGRT | No | 43 | (81) | 9 | (64) | 0.385 | ≈ 0 |
|  | Yes | 10 | (19) | 5 | (36) |  |  |
| RT break | ≤1 week | 48 | (91) | 13 | (93) | 0.083 | ≈ 0 |
|  | >1 week | 5 | (9) | 1 | (7) |  |  |
| AJCC staging manual version | 7^th^ edition | 35 | (66) | 12 | (86) | 0.473 | ≈ 0 |
|  | 8^th^ edition | 18 | (34) | 2 | (14) |  |  |

*AJCC* American Joint Committee on Cancer, *BMI* body mass index, *IGRT* image-guided radiotherapy, *LN* lymph node, *PSW* propensity score weighting, *RT* radiotherapy, *SD* standard deviation.

^†^Rounded.

Table S5. Patient characteristics of the cN1-3 subgroup in SA-6

|  |  | High RT dose  (n=377) | | Low RT dose  (n=200) | | Standardized difference^†^ | |
| --- | --- | --- | --- | --- | --- | --- | --- |
|  |  | Number or mean (SD)^†^ | (%)^†^ | Number or mean (SD)^†^ | (%)^†^ | Before  PSW | After  PSW |
| Age |  | 55.10 (7.95) |  | 57.11 (8.39) |  | 0.245 | ≈ 0 |
| Gender | Female | 23 | (6) | 12 | (6) | 0.004 | ≈ 0 |
|  | Male | 354 | (94) | 188 | (94) |  |  |
| Residency | Nonnorth | 265 | (70) | 54 | (27) | 0.961 | ≈ 0 |
|  | North | 112 | (30) | 146 | (73) |  |  |
| BMI |  | 22.29 (3.58) |  | 22.58 (3.34) |  | 0.083 | ≈ 0 |
| Alcohol use | No | 55 | (15) | 35 | (18) | 0.079 | ≈ 0 |
|  | Yes | 322 | (85) | 165 | (82) |  |  |
| Smoking | No | 47 | (12) | 26 | (13) | 0.016 | ≈ 0 |
|  | Yes | 330 | (88) | 174 | (87) |  |  |
| Clinical stage | 1–2 | 32 | (8) | 5 | (3) | 0.265 | ≈ 0 |
|  | 3–4A | 345 | (92) | 195 | (97) |  |  |
| Clinical T-stage | T1–T2 | 54 | (14) | 9 | (5) | 0.341 | ≈ 0 |
|  | T3–T4 | 323 | (86) | 191 | (95) |  |  |
| Tumor location | Noncervical | 375 | (99) | 197 | (98) | 0.097 | ≈ 0 |
|  | Cervical | 2 | (1) | 3 | (2) |  |  |
| Tumor size | ≤5 cm | 175 | (46) | 93 | (47) | 0.002 | ≈ 0 |
|  | > 5 cm | 202 | (54) | 107 | (53) |  |  |
| Number of LNs examined |  | 22.96 (12.55) |  | 36.22 (17.56) |  | 0.869 | ≈ 0 |
| Use of IGRT | No | 295 | (78) | 146 | (73) | 0.122 | ≈ 0 |
|  | Yes | 82 | (22) | 54 | (27) |  |  |
| RT break | ≤1 week | 353 | (94) | 198 | (99) | 0.288 | ≈ 0 |
|  | >1 week | 24 | (6) | 2 | (1) |  |  |
| AJCC staging manual version | 7^th^ edition | 266 | (71) | 169 | (84) | 0.339 | ≈ 0 |
|  | 8^th^ edition | 111 | (29) | 31 | (16) |  |  |

*AJCC* American Joint Committee on Cancer, *BMI* body mass index, *IGRT* image-guided radiotherapy, *LN* lymph node, *PSW* propensity score weighting, *RT* radiotherapy, *SD* standard deviation.

^†^Rounded.

Table S6. Patient characteristics of the study population in SA-7

|  |  | High RT dose  (n=296) | | Low RT dose  (n=179) | | Standardized difference^†^ | |
| --- | --- | --- | --- | --- | --- | --- | --- |
|  |  | Number or mean (SD)^†^ | (%)^†^ | Number or mean (SD)^†^ | (%)^†^ | Before  PSW | After  PSW |
| Age |  | 54.43 (8.34) |  | 56.70 (8.43) |  | 0.271 | ≈ 0 |
| Gender | Female | 19 | (6) | 12 | (7) | 0.012 | ≈ 0 |
|  | Male | 277 | (94) | 167 | (93) |  |  |
| Residency | Nonnorth | 216 | (73) | 44 | (25) | 1.106 | ≈ 0 |
|  | North | 80 | (27) | 135 | (75) |  |  |
| BMI |  | 22.33 (3.58) |  | 22.44 (3.48) |  | 0.030 | ≈ 0 |
| Alcohol use | No | 43 | (15) | 31 | (17) | 0.076 | ≈ 0 |
|  | Yes | 253 | (85) | 148 | (83) |  |  |
| Smoking | No | 40 | (14) | 25 | (14) | 0.013 | ≈ 0 |
|  | Yes | 256 | (86) | 154 | (86) |  |  |
| Clinical stage | 1–2 | 59 | (20) | 13 | (7) | 0.376 | ≈ 0 |
|  | 3–4A | 237 | (80) | 166 | (93) |  |  |
| Clinical T-stage | T1–T2 | 53 | (18) | 6 | (3) | 0.486 | ≈ 0 |
|  | T3–T4 | 243 | (82) | 173 | (97) |  |  |
| Clinical N-stage | N0 | 34 | (11) | 11 | (6) | 0.189 | ≈ 0 |
|  | N1-N3 | 262 | (89) | 168 | (94) |  |  |
| Tumor location | Noncervical | 294 | (99) | 176 | (98) | 0.093 | ≈ 0 |
|  | Cervical | 2 | (1) | 3 | (2) |  |  |
| Tumor size | ≤5 cm | 136 | (46) | 81 | (45) | 0.014 | ≈ 0 |
|  | > 5 cm | 160 | (54) | 98 | (55) |  |  |
| Number of LNs examined |  | 23.82 (13.34) |  | 36.65 (17.54) |  | 0.823 | ≈ 0 |
| Use of IGRT | No | 235 | (79) | 127 | (71) | 0.196 | ≈ 0 |
|  | Yes | 61 | (21) | 52 | (29) |  |  |
| RT break | ≤1 week | 275 | (93) | 176 | (98) | 0.267 | ≈ 0 |
|  | >1 week | 21 | (7) | 3 | (2) |  |  |
| AJCC staging manual version | 7^th^ edition | 296 | (100) | 179 | (100) | . | . |
|  | 8^th^ edition | 0 | (0) | 0 | (0) |  |  |
| Minimally invasive esophagectomy | Without | 77 | (26) | 9 | (5) | 0.606 | ≈ 0 |
|  | With | 219 | (74) | 170 | (95) |  |  |

*AJCC* American Joint Committee on Cancer, *BMI* body mass index, *IGRT* image-guided radiotherapy, *LN* lymph node, *PSW* propensity score weighting, *RT* radiotherapy, *SD* standard deviation.

^†^Rounded.
